# Supplementary material for: Three weeks of a home-based “sleep low-train low” intervention improves functional threshold power in trained cyclists: A feasibility study
Source: PLoS One. 2021 Dec 2;16(12):e0260959. doi: 10.1371/journal.pone.0260959 (PMC8639084; doi:10.1371/journal.pone.0260959)
Supplement: Attachment — Submitted filename: Response to Reviewers.docx [file pone.0260959.s002.docx]

**Response to Editor:**

Dear Dr. Cosme F. Buzzachera

Thank you for the opportunity to respond to the reviewers’ comments who have provided a fair, thorough, and constructive review of our manuscript. We are pleased that reviewers consider our manuscript well-written, timely and original that provides valuable findings for athletes and scientists. The comments provided by each review represent vastly different feedback on our manuscript and we have aimed to strike an appropriate compromise between reviewer’s comments. We have attempted to address the comments raised by the reviewers and now consider our manuscript to 1) address concerns related to power meter utilisation, and reliability of data reported 2) clarify methodological reporting of subject characteristics and measures 3) clearly highlight the practical implications of the data provided within the manuscript. We hope that the reviewers now consider our revised manuscript worth of publication.

Best Regards

Julien

**Response to Reviewers: Reviewer 1**

The authors are commended for a well-written manuscript. The arguments for the manuscript under review are timely and original. There are, however, significant concerns and methodological issues with the manuscript in its current form, and in my view, the document is unsuitable for publication. The fundamental flaw is the use of diverse, non-scientifically validated power meters. This situation is valid in practical terms but not adequate from a rigid, scientific perspective. All my comments are included below. I hope you will find them to be constructive and helpful.

We are thankful to this reviewer for their thorough and constructive review and are grateful for their comments, all of which have improved this manuscript. We are pleased that the reviewer feels that this piece of work is well-written, timely and original. We have attempted to address all the comments and hope the reviewer now considers the revised manuscript worthy of publication.

The primary concerns with the manuscript are presented below:

1. An unfortunate aspect of the study is the use of multiple, non-scientifically validated (not all!) power meters. This fundamental flaw is vital since power output (PO) data are crucial for the current findings. Although the authors have reported the manufacturer's claimed power meter accuracy, it is not valid from a rigid, scientific perspective. Both validity and reliability of an ergometer/power meter are necessary within a scientific context. Why? Variability within an ergometer (or similar) could arise from both systematic and random error sources. Both error sources, for example, limit our ability to compare repeated tests over time or track changes in physiological outcomes (for details, please see Hopker et al., 2010). Thus, were the improvements in FTP derived from the nutritional intervention per se or related to systematic/random error sources of such devices? Please comment. Lastly, if the calibration was done (lines 204-206), was it done using a dynamic calibration rig - or similar? Please explain.

We would like to thank the reviewer for highlighting their concerns with regards to the power meters used and the reliability of our current testing battery. We have addressed these concerns in multiple ways to best address the level of concern.

1. We have added additional information to Additional File 1 and relocated to the Methods section to outline the distribution of subject’s power meter and the associated literature in support of each device’s validity and reliability (Table 2). Of the devices used by our subjects, the only non-validated device is the TACX Neo, despite this it is frequently used within applied sport and exercise science literature (Lundby *et al.,* 2021, *Journal of Science and Medicine in Sport*; Sitko *et al.,* 2020, *Hospital Nutrition;* Oberholzer *et at.,* 2019, *Frontiers in Physiology*; Pill *et al.,* 2019, *Int J Environ. Reas. and Public Health*) in combination with other devices such as a LODE ergometer (De Korte *et al.,* 2021, *Sports Medicine*)
2. As authors, we aimed to mitigate the concerns with regards to the validity and robustness of the data presented given the use of multiple devices. Therefore, prior to the commencement of the study, all subjects completed a short test-retest reliability study to ensure suitable reliability within the testing battery before continuing with the study. The data was previously intended to be used in an alternative publication however, upon reflection and having considered the concerns of reviewer #1, we have decided to include this data within the present manuscript to support the data within and the subsequent conclusions drawn (Table 4). For ease of comparison, our main reliability metrics have been reported against current laboratory-based reliability studies using exercise durations of a similar duration (see below). We have also highlighted our strong reliability data against laboratory-based measures within the discussion (lines 559-563)
3. We have provided further information with regards to how power meters were calibrated by subjects dependent on the type of device used. Lines 222-224.

| **Paper** | **Test** | **CV (%)** | **TEM (W)** | **ICC** | ***N*** |
| --- | --- | --- | --- | --- | --- |
| Present Study | 20 min PPO | 1.52 | 4 | 0.994 | 55 |
|  | 5 min PPO | 1.4 | 5 | 0.994 |  |
|  | 1 min PPO | 2.36 | 16 | 0.989 |  |
| McGrath *et al., “Is the FTP test a reliable, reproducible and functional assessment tool in highly trained athletes?” (2019)* | 20 min PPO | - | - | 0.980 | 19 |
| MacInnis *et al., “The Reliability of 4-min and 20-min Time Trials and Their Relationships to Functional Threshold Power in Trained Cyclists.” (2019)* | 20 min PPO | - | 5 | 0.99 | 8 |
|  | 4 min PPO | - | 8 | 0.98 |  |
| Borszcz *et al., “Reliability of the Functional Threshold Power in Competitive Cyclists” (2020)* | 20 min PPO | 2.9 | 7 | 0.97 | 25 |
|  | 5 min PPO* | 5 | 13 | 0.93 |  |
| Lillo-Bevia *et al., “Is the Functional Threshold Power a Valid Metric to Estimate the Maximal Lactate Steady State in Cyclists?”* | 20 min PP | 1.2 | - | 0.97 | 11 |
| * Data collected during warm-up | | | | | |

4. Some sample characteristics were poorly reported. For example, were the participants in good health or users of any supplement and/or medication that could affect their metabolism - and indirectly, performance? Also, how were the participants recruited? This kind of information is crucial for the readers. Please insert. Lastly, the inclusion of sample size calculation is also essential for the reader. Is the actual sample (n = 55) enough to explore the hypotheses of the current investigation (I believe yes!)? If not, the reduced sample size should be acknowledged as a limitation. Please insert sample size calculation.

We thank the reviewer for highlighting our inadvertent omission of some important sample characteristics. We have included information outlining the exclusion of subjects in poor health, with metabolic disease or currently taking metabolism altering medications. Information with regards to how subjects were recruited (online, social media recruitment drive) and sample size calculations have been added.

The participants were in good health and not users of any supplement and/or medication that could affect their metabolism – Information added lines 118-119.

Information added related to subject recruitment strategy. lines 108-109.

Sample size calculation indicated a minimum of 16 subjects to detect a 5% difference between groups at 95% statistical power. Information added lines 109-110.

5. Session RPE was assessed by the 0–10 Borg RPE scale. This relatively simple scale, however, requires a multitude of procedures, including the standard definition of perceived exertion and memory- and/or exercise-anchoring procedures (for details, see Noble & Robertson, 1996). Additionally, to define the assessment time following a training session or test (15-min, 30-min, 1-h, 2-h,...) is also essential. Therefore, the lack of these RPE-related procedures should be acknowledged as a limitation.

We thank reviewer one for the comments relating to the measure of RPE and the timeframe after exercising it was recorded. We have added references to support the validity of the scale during exercise and clarified when participants were asked to record this information. Lines 241-245.

6. The authors defined HR as an outcome of interest. However, some pieces of information are still unclear. For example, how was HR monitored? Please specify (Polar, Garmin, Suntuu,...). Importantly, what means "mean HR"? HR is highly variable during a training session and depends on multiple factors other than the effort per se (emotional stress, body temperature, hydration status, use of medications, and others). Hence, the "mean" HR may not reflect the physiological strain of a session training. In addition, how was HRmax estimated or measured? Please inform.

We thank the reviewer for highlighting his concerns with regards to heart rate measurement during exercise. We agree that heart rate is highly variable, and therefore only a secondary variable of interest. Furthermore, we have endeavoured to remove as many contributing factors including the exclusion of participants with metabolic disease, the use of metabolism altering medication alongside limitation of caffeine and alcohol intake, the advice to exercise in a well hydrated state and at the same time each day for those participating in the study. This information has been communicated throughout the methods section. Information has been included with regards to heart rate strap usage, brands/models (Lines 219-220). HRmax calculation and reference has also been added to the methods section (lines 220-221).

Due to the remote nature of the present study, heart rate represented the only physiological metric we were able to collect during exercise. For this reason, we feel it is important to provide physiological context to the current data, especially when power output was reduced during exercise with low carbohydrate availability. Despite power output being reduced, heart rate remained unchanged between groups highlighting comparable physiological workload despite reductions in workload.

7. Although the Ethics committee has approved the study, the authors should consider the risks of the proposed study protocol. Today, exercise physiology labs worldwide are spending much money to increase the safety of both evaluators and volunteers. In the current study, however, each volunteer was asked to perform three heavy tests without any in loco supervision. I understand that this lack of supervision is related to the pandemic and that the participants were "trained" adults. However, adverse events may occur occasionally - we have a recent example in the Euro soccer championship -, and supervision is fundamental. In other words, this lack of in-person supervision in such heavy tests - or proposed training - may lead the participants to "unnecessary" risks. Therefore, sentences such as "Using technology to facilitate exercise monitoring, prescription, and testing, we have shown the potential for data collection away from traditional laboratory-based testing and supervision", should be avoided. Please consider.

We thank the reviewer for his comment and justified concerns with regards to exercising alone and whether this should be advocated for or against. Unfortunately, due to the timing of the present study, lack of supervision was necessitated by the pandemic. To ensure safety of all involved, risks were clearly outlined to the ethics committee; all appropriate risk assessments were included in our application, and this was judged to have minimal risk to the subject group. The risks of the study were also clearly communicated to the participants prior to their agreement to participate.

Our statement "Using technology to facilitate exercise monitoring, prescription, and testing, we have shown the potential for data collection away from traditional laboratory-based testing and supervision" remains valid and we do not advocate excessive risk or training alone/unsupervised. We developed this point further to highlight the necessity of testing in a laboratory-based environment. We have added “Despite this, the best approach for research-centric interventions may be to combine online training and nutritional prescription, with laboratory-based testing protocols to ensure optimal reliability and ensure subject safety during extreme exercise tests” Lines 625-626

8. The purpose of the study was "to assess the feasibility of a 3-week home-based “sleep low-train low” programme and its effects on cycling performance in trained athletes". On most occasions (including the title!), however, the authors reinforce the importance of the COVID-19 period. Why? There is, in my view, not necessary. The current findings could be extrapolated to any context where "outdoor" cycling training is not possible - including, but not exclusive to, the COVID-19 pandemic. The authors are encouraged, therefore, to avoid "highlighting" this specific scenario.

We thank the review for this comment and agree that the study has merit outside of COVID-19 pandemic and has greater application than COVID alone. We have removed references to COVID from the title and from all but essential points within the discussion where COVID represented a significant challenge to data collection and limited our methodological decision making.

9. The study hypothesis is a bit unclear. The authors hypothesized that "performance will be improved to a greater extent in the SL group compared to control conditions". However, the authors also hypothesized that "in the absence of live supervision, adherence will remain high, with only those with 100% completion considered for further analysis". The authors did not measure adherence since they merely decided to include participants who completed all training sessions. Please exclude this final sentence.

Thank you to the reviewer for highlighting this oversight on our part. This hypothesis represented a previous hypothesis prior to the exclusion of non-adherent subjects. It has now been removed.

10. Some pieces of information exist but are still unclear. For example, were the participants asked to avoid caffeinated or alcoholic products and vigorous exercise before (24 h) the assessments? If yes, please inform.

Information added. Lines 150-151.

11. The Discussion section is long and exceptionally speculative, with arguments that are out of the scope of the study. An example is paragraph 3. The authors stated that "considering the acute molecular responses to training with low CHO availability, and the improvements in mitochondrial efficiency following the chronic application of “train low”, it is justified to speculate improvements in muscle oxidative capacity leads to increased performance following a period of low CHO training"; however, it is hard to speculate that this short-time of home-based training intervention improved oxidation muscle oxidative capacity w/o any direct muscle measurement. Therefore, the authors are recommended to only focus on the current findings.

We thank the reviewer for this comment and appreciate that the discussion is long and draws heavily on molecular evidence supporting the notion of improved skeletal muscle adaptation in response to training with low carbohydrate availability. Throughout our introduction we introduce the role of muscle glycogen as a mediator of cellular adaptation and set out the molecular rationale for training with low carbohydrate availability. We suggest the sleep low strategy may be the optimal approach as CHO availability more closely reflects CHO demand during exercise. Having successfully implemented the sleep low strategy in a large cohort of trained cyclists, showing improved performance in the SL group only, we aim to develop the rationale set out within the introduction to support our findings. Furthermore, the length of the discussion was necessitated by the divergent performance responses between the SL and CON groups. We proposed two alternative rationales for an increased performance in 20 min PPO in the SL group and impaired performance in the 1 min PPO.

In the absence of invasive biological samples and data, as authors, we have provided a scientifically justified, well reasoned rationale for the enhanced endurance performance observed in the sleep low condition. We have drawn upon the collective expertise of the authors and extensive literature from our, and collaborators labs, we have explained the differences in performance between groups. In the absence of speculation, and based on the current findings, there is no obvious approach to rationalising the present data.

We have also highlighted that despite the relatively short intervention duration, several other studies have shown beneficial responses to training with low CHO compared to high CHO ranging from 2-4 weeks (Cochran *et al.,* 2015; Marquet *et al.,* 2016; Riis *et al.,* 2019. Our results are in line with evidence from laboratory-based interventions.

**Response to Reviewer #2:**

Overview
The subject is interesting and has a lot of relevance, especially at this pandemic moment.
It has great applicability and shows the benefit of using remote monitoring systems.
I did some commentaries in the text (PDF file).

We would like to thank the reviewer for their overwhelmingly positive feedback and for considering our manuscript relevant with practical application.

Introduction

1. At the final of this section, it seems to be a scientific gap, but it´s not clear. Please revise and insert the scientific gap of the study.

As suggested, we have amended the final section of the introduction.

Discussion

2. As reviewer I suggest rewriter this paragraph follow the suggestions:

1) Start mention the objective of the study;

2) Cite the main findings;

3) Write if the main findings are in accordance or not with the hypothesis.

We thank the reviewer for their comments and have amended the first paragraph to better reflect the suggested layout.

3. Before conclusion I suggest insert clearly the practical application because the most important contribution of this study, in my point of view, is the applicability and benefit of using remote monitoring systems especially in this moment of pandemic, as a tool for monitoring distance training, generating reliable results and making athletes and practitioners not feel alone in relation to their training.

We thank the reviewer for this comment and agree that for a paper of this nature, it is important to clearly highlight the practical applications of this study. As such we have added a “Practical applications” section prior to the conclusion.
